# Pain Reduction With an Immersive Digital Therapeutic Tool in Women Living With Endometriosis-Related Pelvic Pain: Randomized Controlled Trial

**DOI:** 10.2196/39531

**Published:** 2022-09-21

**Authors:** Benjamin Merlot, Garance Dispersyn, Zoé Husson, Isabella Chanavaz-Lacheray, Thomas Dennis, Juliette Greco-Vuilloud, Maxime Fougère, Stéphane Potvin, Maryne Cotty-Eslous, Horace Roman, Serge Marchand

**Affiliations:** 1 Franco European Multidisciplinary Endometriosis Institute (IFEMEndo) Bordeaux France; 2 Lucine Bordeaux France; 3 Centre de Recherche de l'Institut Universitaire en Santé Mentale de Montréal Montréal, QC Canada; 4 Faculté de Médecine et des Sciences de la Santé Centre de Recherche Clinique du Centre Hospitalier Universitaire de Sherbrooke Université de Sherbrooke Sherbrooke, QC Canada

**Keywords:** digital treatment, digital intervention, virtual reality, pelvic pain, endometriosis, digital therapeutics, chronic pain, randomized controlled trial, RCT, pain, women's health, eHealth, digital health, endometrium, pelvis, pelvic, efficacy, effectiveness, gynecology, gynecologist, sexual health, reproductive health

## Abstract

**Background:**

Chronic pelvic pain is a common and disabling condition in women living with endometriosis. Pharmacological and surgical treatments are not always effective at controlling pain and present important restrictions. Digital therapeutics (DTx) are emerging as major nonpharmacological alternatives that aim to extend the analgesic therapeutic arsenal of patients.

**Objective:**

In this randomized controlled trial (RCT), we aimed to measure the immediate and 4-hour persisting effects of a single use 20-minute DTx (Endocare) on pain in women experiencing pelvic pain due to endometriosis.

**Methods:**

A total of 45 women with endometriosis participated in a randomized controlled study comparing the analgesic effect of a single use of a virtual reality digital treatment named Endocare (n=23, 51%) to a 2D digital control (n=22, 49%). Perceived pain and pain relief were measured before the treatment and 15, 30, 45, 60, and 240 minutes after the end of the treatment.

**Results:**

The clustered posttreatment pain was significantly reduced compared to the pretreatment for both Endocare and the control group (all *P*<.01). Endocare was significantly more effective than the control group (all *P*<.01). Endocare decreased the mean pain intensity from 6.0 (SD 1.31) before the treatment to 4.5 (SD 1.71) posttreatment, while the control only decreased it from 5.7 (SD 1.36) to 5.0 (SD 1.43). When comparing each posttreatment measures to the pretest, Endocare significantly reduced pain perception for all points in time up to 4 hours posttreatment. The differences did not reached significance for the control group. Moreover, Endocare was significantly superior to the control group 15, 30, and 45 minutes after the treatment (all *P*<.001). The mean perceived pain relief was significantly higher for Endocare at 28% (SD 2%) compared to the control, which was 15% (SD 1%) for all the posttreatment measurements (all *P*>.05).

**Conclusions:**

Our study aimed to test the effects of a single use of a DTx treatment on reported pain at different time points in women diagnosed with endometriosis experiencing moderate-to-severe pelvic pain. Importantly, our results support that Endocare, a virtual reality immersive treatment, significantly reduce pain perception compared to a digital control in women living with endometriosis. Interestingly, we are the first to notice that the effect persisted up to 4 hours posttreatment.

**Trial Registration:**

ClinicalTrials.gov NCT04650516; https://tinyurl.com/2a2eu9wv

## Introduction

Endometriosis is characterized by lesions occurring outside the uterus whose appearance and behaviors are close to those of the endometrium mainly on the pelvic peritoneum, ovaries, and rectovaginal septum [1]. It might also reach deeper tissues, such as the rectum, bladder, ureters, colon, small bowel, diaphragm, or pelvic nerves. Endometriosis is a multifactorial disease resulting from the combined action of genetic and environmental factors [2,3] and is a major contributor to chronic pelvic pain (CPP) and infertility among women [4].

The mean prevalence of endometriosis in women with CPP has been estimated at 70% (SD 3%) [5], but it may vary from 2% to 93% depending on the study [5,6]. CPP is the main symptom of women with endometriosis [7]. The most common painful symptoms of endometriosis are dysmenorrhea and deep dyspareunia, appearing in nearly 80% and 30% of patients, respectively [1]. The pain, which can severely affect quality of life, can be constant or triggered by various conditions, such as menstruation [8].

Current management of endometriosis pain involves pharmacological (eg, hormonal therapy) and surgical treatments [9-11]. The goals of these medical therapies are multifold (eg, reduction of inflammation, inhibition of ovulation, suppression of menstruation) [1]. These are based on the concept that the response of the eutopic endometrium and endometriosis lesions is substantially similar.

Hormonal therapies or surgeries are not adapted for women willing to get pregnant. Therefore, the development of nonpharmacological alternatives for the management of endometriosis pain is critical to extend the treatment arsenal for women with pelvic-perineal pain and endometriosis [12]. Among them, pilot evidence asserts the benefits of mindfulness training (eg, meditation, breathing, music) on pain relief [13]. Various levels of quality of life (ie, physical, mental and social features) are affected in women living with endometriosis [14,15]. Therefore, additions to the treatment of psychological interventions (eg, hypnotherapy, cognitive behavioral therapy) have shown significant results for both physical and mental well-being in patients living with CPP and endometriosis [16,17].

In the past few decades, the emergence of digital therapeutics (DTx) aimed at the use of informatic tools to aid the diagnosis and therapy of various pathologies has led to the creation of many new therapeutic devices [18]. Recently, multiple meta-analyses have specifically supported the efficacy of virtual reality (VR) in several type of acute and chronic pain conditions [19-24].

On these bases, a new digital therapeutic approach, Endocare, was created by combining various therapeutic procedures based on several modalities in a VR environment, individually known to reduce pain [25-27]. Endocare therapeutic procedures comprise auditory (eg, alpha/theta binaural beats, nature-based sounds) and visual (eg, bilateral alternative stimulations) components associated with a 3D VR environment. To our knowledge, no studies have ever tested the effects of this type of nonpharmacological treatment on reported pain at different time points in patients diagnosed with endometriosis perceiving moderate-to-severe pelvic pain. In this randomized controlled trial (RCT), we hypothesized that a single use of the Endocare treatment would be able to significantly diminish the pain intensity in patients diagnosed with CPP associated with endometriosis.

## Methods

### Design and Setting

This was a randomized, controlled, comparative, open-label, 2-parallel-group interventional study comparing the effect of Endocare and a digital control on endometriosis-related pain after a single use. This RCT was conducted between December 2020 and May 2021 at the Franco-European Multidisciplinary Endometriosis Institute (IFEMEndo), Clinic Tivoli-Ducos in Bordeaux, France.

### Ethics Approval

This study was conducted in compliance with good clinical practice guidelines, the principles of the Declaration of Helsinki, and French laws and regulations. It was reviewed and approved on November 6, 2020, by the Comité de Protection des Personnes. All participants completed and signed the informed consent form before inclusion in the study and before any study-related procedure began. Before commencing participant enrollment, this study was registered on ClinicalTrials.gov (NCT04650516).

### Quality Assurance

Standard operating procedures were applied for the conduct and analysis of the clinical investigation. This study was monitored regularly according to the specifically designed monitoring plan. Furthermore, data were captured following the double entry procedure in the dedicated case report form and compared. Discrepancies were reviewed and corrected by a third entry clerk. A data validation document specifically written for this study aimed to list all data checks to be performed. A data manager programmed the checks with Ennov Clinsight, and the sponsor validated the checks. In case of inconsistencies, a query was edited during the quality analysis beyond data entry. The investigator or another authorized person from the clinical staff was asked to answer the query by confirming or correcting the data.

### Population

#### Selection Criteria

The selection criteria were women over 18 years old with a magnetic resonance imagery (MRI) diagnosis of endometriosis who were willing to participate in the study and signed the informed consent form. All patients were recruited from a highly specialized center (ie, IFEMEndo). These patients experienced mostly severe pain related to their chronic pain condition and presented a long medical history concerning their endometriosis, which was often deep endometriosis.

#### Inclusion Criteria

To be included in this study, the screened participants who met the selection criteria had to be living with moderate-to-severe endometriosis-related pain with a score ≥4 on an 11-point numerical rating scale (NRS) at the time of inclusion, a criterion shared among various studies [8].

#### Exclusion Criteria

The exclusion criteria were women who (1) were pregnant or breastfeeding; (2) had consumed painkillers within 8 hours prior to inclusion; (3) were participating in an interventional study or had participated in an interventional study within 30 days before enrolment; (4) were employed by the investigator or study site, with direct involvement in the proposed study or other studies under the direction of that investigator or study site, as well as family members of the employees or the investigator; and (5) had a contraindication to Endocare or the digital control, such as severe visual, hearing or cognitive impairments, color blindness, photosensitivity, epilepsy, or motion sickness.

#### Sample Size Determination

This study aimed to evaluate the short-term pain evolution after a single use of Endocare compared to a digital control in participants experiencing endometriosis-related pelvic pain. The primary end point was the mean pain intensity 60 minutes after the beginning of treatment (or last evaluation if the participant dropped out of the study or started any rescue medication). Gerlinger et al [28] determined that women who felt “minimally satisfied” in the management of their pain had a change of −19.5 mm (SD 14.3 mm) on a visual analogical scale. Hence, a 20-mm difference between Endocare and the digital control seemed like a reasonable clinical target and was taken into consideration for sample size calculation. We planned to measure a total of 40 participants, with 20 allocated to the intervention group and 20 to the control group, at 5 time points.

With a 2-sided 95% CI, the study achieved 81% power to detect a difference between the group means at the last time of 20 mm on a 100-mm VAS with a 25-mm standard deviation. The correlation between measurements within a participant was estimated at 0.500. A test based on a mixed-model analysis was anticipated at a significance level of 5%. Assuming that about 25% of women would not experience an endometriosis-related pain ≥4 on NRS at time of inclusion, we planned to screen 50 women. Consequently, at least 40 women were planned to be included in the study.

Finally, 46 participants were screened, of which 45 were included and randomized; 1 was a screen failure because she never went back for the study visit (Table 1). A total of 44 (97.78%) participants completed the entire study.

**Table 1:** Size of the sample.

Sample	Value
Participants screened, n	46
Participants randomized, n (%)	45^a^ (100)
Participants who completed study, n (%)	44^b^ (97.8)

^a^One participant was lost to follow-up between the screening visit and the randomization visit and their inclusion criteria could not be checked.

^b^The self-administered questionnaire of 1 participant was never received for analysis.

### Concomitant Medications

Pain medications were stopped at least 8 hours prior to participation. If pain persisted after treatment (Endocare or control), the patient could take their pain management rescue treatment, thus ending the data collection at that time.

### Treatment and Control

#### Endocare

**Figure 1:**
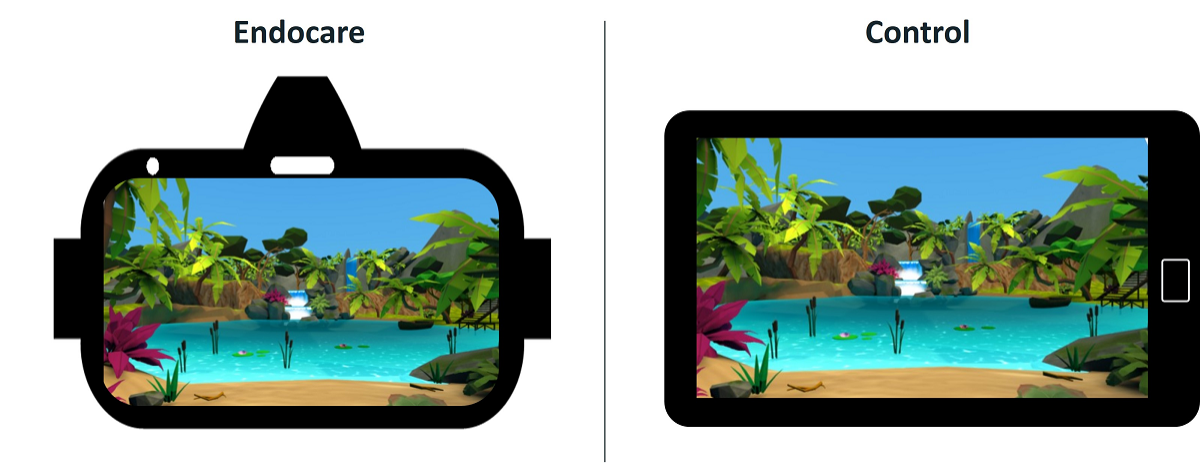
Visual display of the 3D virtual reality treatment (ie, Endocare) and the 2D tablet control.

We developed the Endocare treatment (Lucine, Bordeaux, France) specifically for this study (Figure 1). It was displayed through a VR headset (Oculus Quest) with high-quality headphones (APK K-240-MKII). Endocare is a standalone medical software device comprised of an application stored in a VR headset that is intended to mitigate pain for people prone to endometriosis. Endocare offers a 20-minute treatment consisting of a combination of auditory (eg, alpha/theta binaural beats, nature-based sounds) and visual (eg, bilateral alternative stimulations consisting of a sphere appearing and moving on a horizontal axis) therapeutic procedures integrated in a 3D VR environment.

#### Control

The digital control was also developed by Lucine specifically for this study (Figure 1). The digital control program was displayed through a tablet (Samsung Galaxy Tab A) with high-quality headphones similar to those used in the Endocare group (APK K-240-MKII). The digital comparator was a 20-minute control with the same composition as the Endocare treatment (same context, environment, and duration) but without any immersive effects of the VR headset itself, nor the auditory (eg, alpha/theta binaural beats, nature-based sounds) and visual (eg, bilateral alternative stimulations) stimuli. A soundtrack composed of nature sounds related to the projected image.

### Procedures

Women living with endometriosis pain were recruited from IFEMEndo*,* a clinic that specializes in endometriosis. All patients were diagnosed with endometriosis by specialized gynecologists and medical procedures (eg, MRI). Recruitment was done when the patients came for their medical examination and were informed about this study. If the patients expressed interested in participating in this study, the research team offered them a new appointment at the clinic to receive the study treatment.

### On-site Visit

When patients returned to the clinic on the day of the inclusion visit, their pain level needed to be at least 4 on an 11-point numeric pain rating scale to participate in this study. Patients reporting a pain of <4 on this scale were considered as screen failures and withdrawn from the study. This pain intensity evaluation represented the pretreatment evaluation just before the use of Endocare or control and is considered the baseline (T0).

If the patient consented to participate and met the inclusion criteria, they were randomized to 1 of the 2 groups, Endocare or control. The treatment (either Endocare or control) consisted of a single use of the VR headset on the day of the visit, as presented in Table 2.

The research team accompanied the patient to distinct isolated rooms and then handed them either the headset (ie, Endocare) or the tablet (ie, control). Patients were then guided orally by the investigator for up to 5 minutes until the beginning of the treatment (ie, Endocare or control).

**Table 2:** Study flowchart.

Study procedures	Screening	Care and follow-up	Follow-up until 240 minutes after treatment
Informed consent	✓		
Eligibility criteria (without pain assessment)	✓		
Demographics	✓		
History and management of endometriosis	✓		
Typology of pain crises	✓		
Baseline pain assessment (T0) (just before the start of Endocare or control treatment)		✓	
Assessment of general status	✓	✓	✓
Endocare or control treatment		✓	
Pain evolution (pain relief and intensity)		✓^a^	✓^b^
Satisfaction			✓^b^
Concomitant treatments		✓^c^	✓^c^
Adverse events		✓	✓

^a^Evaluation 15 minutes (T15), 30 minutes (T30), 45 minutes (T45), and 60 (T60) minutes posttreatment.

^b^Evaluation 240 minutes (T240) posttreatment.

^c^Includes rescue treatment if needed.

### Follow-up

Once the treatment was completed, each participant was asked to rate on a paper questionnaire their pain perception on an 11-point NRS (0: no pain, 10: unbearable pain) and their perceived pain relief on a 5-point categorical scale 15 minutes (T15), 30 minutes (T30), 45 minutes (T45), and 60 minutes (T60) after administration of the allocated study treatment. During the first hour, participants remained under the direct supervision of the study site staff. After the first hour, and if cleared by the study site staff, participants were free to leave the study site and go home to complete next assessment 240 minutes (T240) after the end of the treatment. Adverse events (AEs), if any, were collected during the entire duration of patients’ participation. Before leaving the clinic, participants underwent a general health status check to ensure they were not experiencing any AEs and were able to go home.

At home, patients were asked to rate on a paper questionnaire their pain relief on a 5-point categorical scale and pain intensity based on the 11-point NRS at T240 or at the last time point if the participant dropped out the study or began taking a rescue medication before the end of the follow-up period.

### Measurements

#### Pain Assessment and Pain Relief

Pain intensity was evaluated on an 11-point NRS at T0, T15, T30, T45, T60, and T240. This scale is the reference tool to assess pain in most clinical trials, and it has been used in various recent studies, including those related to endometriosis [29-31]. Notably, its use is recommended by the IMMPACT (Initiative on Methods, Measurement, and Pain Assessment in Clinical Trials) guidelines [32].

Pain relief was evaluated on a 5-point categorical scale (0: no relief, 1: slight relief, 2: moderate relief, 3: lots of relief, and 4: complete relief) at T0, T15, T30, T45, T60, and T240.

If rescue medication was needed, the participant was asked to rate their pain intensity or pain relief just prior to the intake of the rescue medication. Study follow-up was terminated upon the intake of rescue medication.

#### Patient Replacement

Patients who dropped out before the end of the 240-minute follow-up period were not replaced.

#### Statistical Analyses

A linear mixed-model framework was used for statistical analysis (SPSS 2020, IBM Corp). The selected covariance matrix of the repeated measurements that better fitted the data based on the Akaike Information Criterion was the first-order ante-dependence. There were 3 explanatory fixed factors: group (Endocare, control), time (T0, T15, T30, T45, T60, T240), and group*time, as well as a random effect on the intercept of each participant. In the first analysis, time (fixed effect) was parametrized as baseline (T0) versus all other times (T15 to T240). The analysis consisted of comparing T0 with the 5 posttreatment data clustered for both groups and looking at the interaction term (group*time). A contrast analysis was also performed to verify the difference between baseline (T0) and posttreatment times (clustered) in the 2 separate groups. In the second analysis, time was parametrized as T0, T15, T30, T45, T60 and T240. A contrast analysis was also performed to test the difference between T0 and all other times (separated) in both groups. Bonferroni corrections were applied for multiple comparisons.

For pain relief, data were analyzed using R software. Because data did not satisfy to normality when assessed with the Shapiro-Wilk normality test, Wilcoxon unilateral unpaired tests corrected for multiple comparisons with a false discovery rate (FDR) were used.

## Results

### Study Participants

Of the 45 women that participated in the study, 1 (2%) did not return the questionnaire. Among the patients included, 1 (2%) from the treatment group and 4 (9%) from the control group took rescue medication after T60 but before T240. Therefore, in accordance with the study design, no results at T240 were collected for these 5 patients. Participants from both groups were comparable in terms of age, height, and weight (Table 3), as well as in pain intensity at T0.

All participants were recruited from a clinic that specializes in endometriosis and were living with severe endometriosis-associated symptoms (Table 4). Of the participants, 90% (n=41) were living with CPP not related to menses, and the majority were living with dysmenorrhea, dysuria, and dyspareunia. Moreover, 77% (n=35) were living with deep infiltrating endometriosis, and 22% (n=10) had adenomyosis.

Most of the patients were taking different classes of medications for their endometriosis condition, including hormones, analgesics, and antidepressants (Table 4). Since this study was based on an add-on protocol, participants were authorized to continue all their medications, except for pain drugs before the beginning of the testing. The participants kept these analgesics as rescue medication after the Endocare or control treatment if needed.

**Table 3:** Participant demographics.

Characteristics		Total (N=45)	Endocare (n=23)	Control (n=22)
**Age (years)**				
	M^a^ (missing^b^)	45 (0)	23 (0)	22 (0)
	Mean (SD)	32.7 (8.02)	32.2 (8.02)	33.2 (8.12)
	Median	31	32	30.5
	Min^c^	21	21	21
	Max^d^	53	53	51
**Height (cm)**				
	M (missing)	44 (1)	22 (1)	22 (0)
	Mean (SD)	162.7 (7.22)	162.6 (6.72)	162.8 (7.84)
	Median	165	165	164
	Min	148	148	150
	Max	175	175	175
**Weight (kg)**				
	M (missing)	44 (1)	22 (1)	22 (0)
	Mean (SD)	63.8 (12.51)	67.2 (12.46)	60.4 (11.88)
	Median	60	63.5	57
	Min	45	50	45
	Max	95	95	92

^a^M: total number of cases.

^b^Missing: total number of cases with missing data.

^c^Min: minimum.

^d^Max: maximum.

**Table 4:** Participants’ history of endometriosis.

Variable			Total (N=45)	Endocare (n=23)	Control (n=22)
**Time since endometriosis diagnosis (months)**					
	M^a^ (missing^b^)		45 (0)	23 (0)	22 (0)
	Mean (SD)		45.2 (54.9)	43.4 (59.3)	47.2 (51.1)
	Median		23.8	16.6	23.8
	Min^c^		2.2	2.5	2.2
	Max^d^		277.8	277.8	199.7
**Type of endometriosis, n (%)**					
	Superficial peritonea		10 (22.2)	6 (26.1)	4 (18.2)
	Deep infiltrating endometriosis		34 (75.6)	17 (73.9)	17 (77.3)
	Digestive locations		1 (2.2)	1 (4.3)	0 (0)
	Other		2 (4.4)	1 (4.3)	1(4.5)
Presence of adenomyosis, n (%)			11 (24.4)	6 (26.1)	5 (22.7)
History of surgical management of endometriosis, n (%)			31 (68.9)	14 (60.9)	17 (77.3)
**Time from endometriosis diagnosis to surgery (months)**					
	M (missing)		31 (14)	14 (9)	17 (5)
	Mean (SD)		40.6 (43.9)	40.5 (36.5)	40.6 (50.2)
	Median		24.4	38.8	23.8
	Min		0.1	2.1	0.1
	Max		199.7	136.0	199.7
**Symptom resolution following surgery, n (%)**					
	Yes		13 (41.9)	3 (21.4)	10 (58.8)
	No		18 (58.1)	11 (78.6)	7 (41.2)
**Time from surgery to symptom recurrence (months)**					
	M (missing)		13 (0)	3 (0)	10 (0)
	Mean (SD)		35.1 (55.2)	9.5 (15.5)	42.8 (61.1)
	Median		21.1	1.7	22.0
**Current management of endometriosis, n (%)**					
	**Hormone-based therapy**				
		None	10 (22.2)	6 (26.1)	4 (18.2)
		Combined hormonal contraceptives	14 (31.1)	7 (30.4)	7 (31.8)
		Progestogens	18 (40.0)	9 (39.1)	9 (40.9)
		Gonadotrophin-releasing hormone agonists	1 (2.2)	0 (0)	1 (4.5)
		Other	2 (4.4)	1 (4.3)	1 (4.5)
	Chronic analgesic treatment		26 (57.8)	14 (60.9)	12 (54.5)
	Other treatment		16 (35.6)	7 (30.4)	9 (40.9)
**Usual pain symptoms, n (%)**					
	Chronic pelvic pain not related to menses		41 (91.1)	21 (91.3)	20 (90.9)
	Dysmenorrhea		24 (53.3)	12 (52.2)	12 (54.5)
	Dysuria		14 (31.1)	6 (26.1)	8 (36.4)
	Dyschesia		1 (2.2)	1 (4.3)	0 (0)
	Dyspaneuria		29 (64.4)	15 (65.2)	14 (63.6)

^a^M: total number of cases.

^b^Missing: total number of cases with missing data.

^c^Min: minimum.

^d^Max: maximum.

### Pain Perception

#### Difference in Pain Perception Between Baseline and the 5 Clustered Posttreatment Measurements

**Figure 2:**
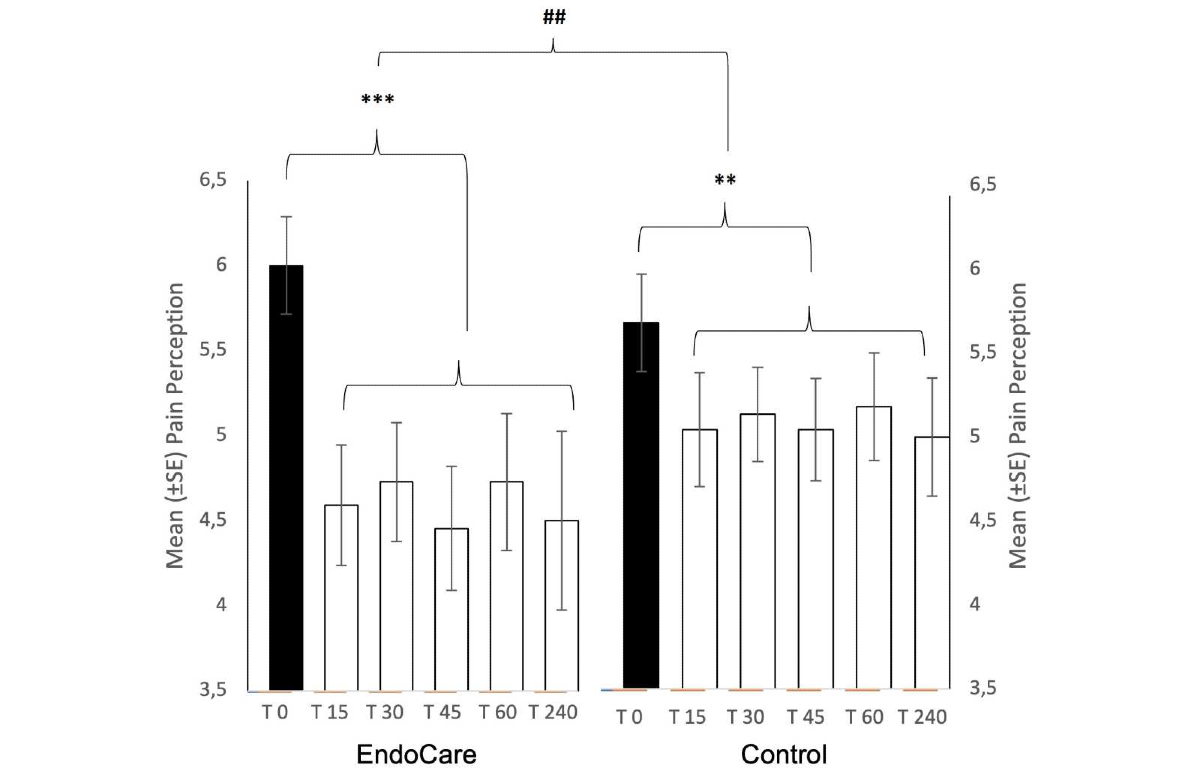
Difference in pain perception between baseline and the 5 clustered posttreatment measurements. Mean pain perception (± standard error) in both groups (ie, Endocare, control) at baseline (black: T0) and the 5 clustered posttreatment measurements (white: T15, T30, T45, T60, and T240). ##*P*<.01 (interaction group*time); **P<.01 (mixed model) ***P<.001 (mixed model).

We first analyzed the differences between the baseline pain (T0) and the 5 clustered posttreatment measurements covering 15 minutes to 4 hours after the treatment (ie, T15, T30, T45, T60, and T240) in both groups using a linear mixed model (Figure 2). We did not observe any effect of the group (Endocare, control; *F_40,712_*=.060; *P*=.807), but we did observe an effect of the time (T0; *F_43,094_*=44.179; *P*<.001). Moreover, we found an interaction for group*time (*F_43,094_*=7.343; *P*=.010), indicating that the mean reduction of pain through time (T0) was significantly different between each group (ie, Endocare, control). Contrast analysis revealed that the mean reduction of pain was greater in the Endocare group (1.58; *t_42,926_*=6.624; *P*<.001) than in the control group (0.38; *t_43,252_*=2.781; *P*=.008).

#### Difference in Pain Perception Between Baseline and Each of the 5 Posttreatment Measurements

**Figure 3:**
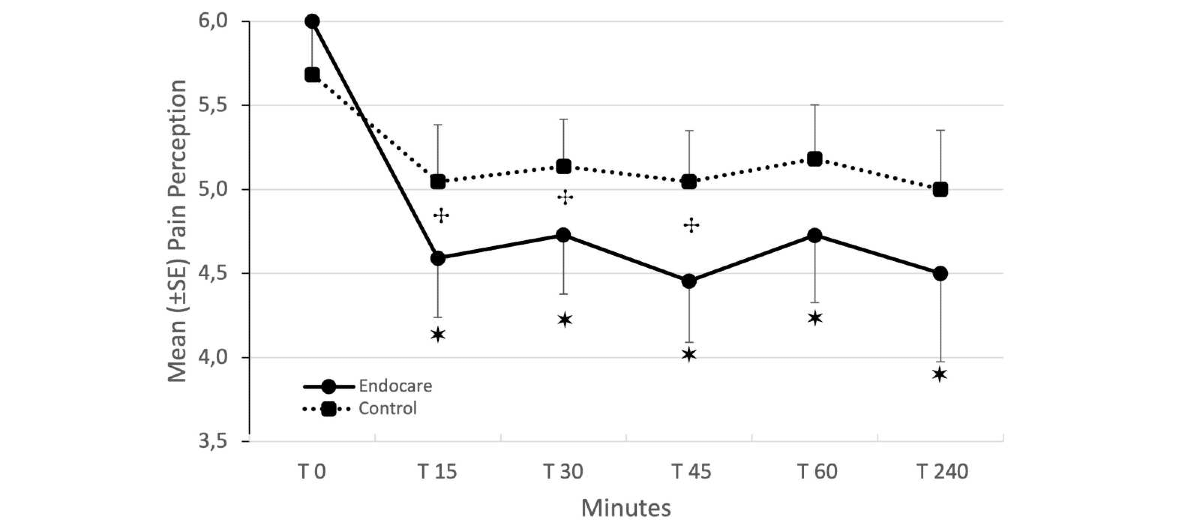
Difference in pain perception before and each of the 5 posttreatments measurements. Mean pain perception (± standard error) in both groups (bold line: Endocare; dash-line: control) at baseline (T0) and each of the 5 posttreatment measurements (T15, T30, T45, T60, or T240). +*P*<.05 (*t* test); *P<.05 (mixed model).

We then aimed to analyze the differences between the baseline (T0) and each of the 5 posttreatment measurements separately (ie, T15, T30, T45, T60, or T240) in both groups using a mixed model (Figure 3). We did not observe any effect of the group (*F_41,122_*=0.716; *P*=.402), but we did observe an effect of the time (*F_57,235_*=10.066; *P*<.001). Moreover, we did not find an interaction of group*time (*F_57,235_*=1.618; *P*=.170), likely due to a weak statistical power. Contrast analysis revealed group*time interactions at T15 (*t_42,000_*=2.211; *P*=.033), T30 (*t_46,241_*=2.226; *P*=.031), and T45 (*t_41,389_*=2.53; *P*=.015), a tendency for T60 (*t_38,964_*=1.946; *P*=.059), and none for T240 (*t_37,872_*=1.618; *P*=.114). This indicates that the mean reduction of pain through time was significantly different between each group (ie, Endocare, control) from T15 to T45, but it was not significantly different from T60 to T240. For the Endocare group, the pain perception reduction was significant in each of the 5 posttreatment measurements (Figure 2, all *P*<.05). For the control group, except for a tendency at T15 (*t_42,000_*=2.576; *P*=.068), no significance was reached relative to the pain perception reduction in each of the 5 posttreatment measurements (all *P*>.05).

#### Covariance For Adenomyosis and Surgery

A total of 31 (70%) participants had surgery related to endometriosis (14 in the Endocare group and 17 in the control), and 11 (25%) suffered from adenomyosis (6 in the Endocare group and 5 in the control). Thus, we introduced 2 covariates, surgery and adenomyosis, into the linear mixed model. Neither surgery (*F_41,800_*=.009; *P*=.924) nor adenomyosis (*F_41,782_*=.001; *P*=.982) changed the results. This indicates that the effect of the treatment on pain was independent of these 2 conditions.

#### Difference in Pain Relief at Each of the 5 Posttreatment Measurements

**Figure 4:**
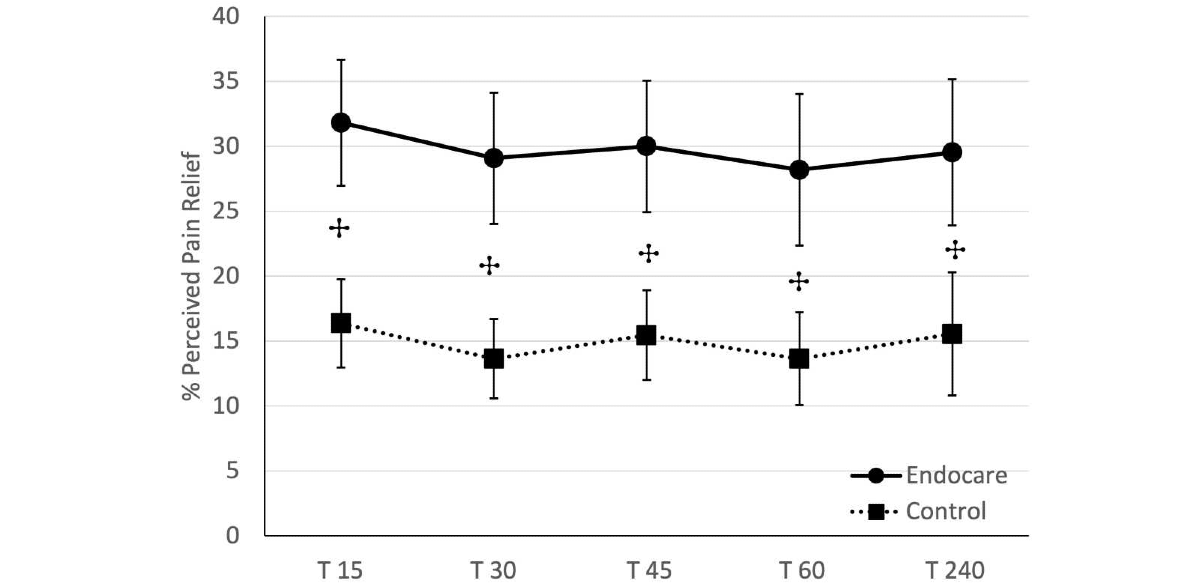
Difference in pain relief at each of the 5 posttreatment measurements. Percentage of pain relief (± standard error of the mean) in both groups (circles and bold line: Endocare; squares and dash-line: control) at each of the 5 posttreatment measurements (T15, T30, T45, T60, or T240). +*P*<.05 (Wilcoxon).

At each posttreatment measurements, participants were asked to measure their perceived pain relief on a 5-point numerical scale from no relief to total relief (Figure 4). The mean pain relief reported was 28% (SD 24.28%) for Endocare and 15% (SD 16.34%) for the control group. Nonparametric Wilcoxon unilateral unpaired tests corrected for multiple comparisons with FDR were performed. All posttreatment measurements present a significantly higher pain relief score for Endocare compared to the control group (all *P*<.05).

#### Maximum Reduction in Pain Assessed by NRS

Finally, we analyzed the maximum reduction of pain intensity in both groups. The mean maximum effect was 42% (95% CI 30.82-53.18) for Endocare and 22% (95% CI 15.38-28.53) for the control group. The maximum effect was significantly higher for the treatment group (Endocare; Cochran *t*-test, *P*=.004).

#### Adverse Events

Seven (15%) participants reported mild-to-moderate AEs, of which 4 (8%) were evaluated as probably unrelated and 3 (6%) as possibly related to the Endocare treatment. The 3 possibly related events were within the treatment group and described as a mild headache and nausea related to motion sickness.

## Discussion

### Principal Results

In this RCT, we aimed to measure the immediate and 4 hours persisting effects of a single use of a 20-minute DTx (Endocare) on pain in women living with pelvic pain related to endometriosis. We showed that Endocare was able to significantly reduce the overall pain perception when comparing the pain at baseline (T0) with the combined 5 posttreatment measurements from 15 minutes (T15) to 4 hours (T240).

We found that our digital control was also able to significantly reduce the overall pain perception but significantly less than the Endocare treatment. Next, we wanted to evaluate whether the pain reduction was significant at each posttreatment measurement (ie, T15, T30, T45, T60, and T240) compared to baseline (T0) in each group. We showed that this was the case for the Endocare group but not for the control group, thus confirming the analgesic effect of the Endocare treatment. To our knowledge, our study is the first to demonstrate an effect on pain reduction in women living with moderate-to-severe pelvic pain due to endometriosis by using a nonpharmacological treatment that combined VR with visual and auditory stimuli.

### Comparison With Prior Work

Numerous studies have shown that VR is an effective way to reduce acute pain intensity, especially pain experienced during medical procedures, by burn victims, or by women during childbirth [24,33-36]. The vast majority of the studies on acute pain only measured pain intensity directly after the treatment, within minutes, or up to 1 hour [34,35,37,38]. Some studies also looked at the effects of VR on chronic pain [24,36]. For instance, studies on patients living with neuropathic, musculoskeletal, phantom limb, or indefinite chronic pain showed reduced pain intensity during or immediately after VR application [39-43]. More recently, a study also reported that 56 days of VR was effective at reducing chronic low back pain throughout the entire duration of the treatment [8]. However, there was no precise indication of how long participants rated their pain after the treatment intake during each of the 56 days. In our study, we measured pain intensity in a short-term period, showing that VR can reduce pain intensity for up to 4 hours, which is comparable to some analgesics. Indeed, a systematic review noted multiple analgesics for their ability to relieve acute postoperative pain by half over a 4-6-hour period [44]. In our study, to potentialize VR effects, we chose various auditory (eg, alpha/theta binaural beats, nature-based sounds) and visual (eg, bilateral alternative stimulations) therapeutic procedures known to relieve pain in different conditions (eg, premenstrual pain, medical imaging procedures, experimental pain, or caesarean section [25-27]). Lucine aimed to combine these stimuli into the DTx treatment Endocare to act on different components of pain. Among the auditory stimuli used in the treatment, binaural beats are associated with a calm and positive affect and are known to reduce stress and anxiety [45,46], contributing to reduced pain perception. Studies also reported that nature-based sounds can promote relief, concentration, and asleep [47], especially by masking environmental noise [48,49] or by amplifying slow waves sleep [50,51]. Therefore, these auditory stimuli can help people with CPP and endometriosis relax and feel reduced pain. Listening to nature sounds is also used in the medical context to reduce pain during surgeries, such as cesarean section, or for intensive care patients [27,52]. Moreover, a systematic review indicated that visual bilateral alternative stimulations can effectively reduce chronic pain [53].

Thus, the combination of these stimulations potentialized with the VR experience could lead to the decrease in pain observed in the Endocare group immediately after and up to 4 hours posttreatment.

In the field of VR research, while some studies do not use a control group [40,42], others compare the effects of VR with medication intake [54] or with totally different controls, such as closing the eyes or a distraction selected by the control group itself (eg, reading, meditating) [35,39,41]. This can be problematic since it is difficult to conclude on the use of VR or a simple headset effect. To our knowledge, only 1 study used a sham VR group, with noninteractive 2D nature scenes, and reported positive outcomes in both groups with reductions in pain and all domains of pain-related interference [8]. In this study, the sham group used the same VR apparatus as the other group. However, the environment of the 2 groups was not identical: the treatment group had a visual display skill-based interactive 3D environment, while the sham group had a noninteractive 2D nature scene. We purport that this type of control is closer to the treatment and thus easier to be double-blinded. Nevertheless, we could not definitively conclude on whether our results were due to the stimulations or the headset itself, since the immersive effect of the headset could be considered as part of the treatment [55].

In this study, we chose to use a digital control program displayed through a 2D tablet with a headset, allowing us to control for the Endocare treatment and not solely the VR apparatus or the stimuli. Indeed, our digital control contained the same composition as the Endocare treatment (ie, context, environment, duration) without the immersive aspect of VR or the auditory and visual stimuli of the treatment. However, to preserve an immersive-like session, we chose to keep a soundtrack composed of nature sounds related to the projected image in the control group, which could potentially explain the positive outcome observed. This slight decrease in pain intensity observed in the control group indicates that our control is relevant.

### Limitations

One limitation of our study may be the population, as we decided to work with patients living with mostly severe pain related to a complex and chronic pain condition, which could represent a selection bias. We likely could have had slightly better treatment results with less severe pain conditions. However, to assess this potential selection bias and analyze whether the beneficial effect would be lessened in populations with complex pathologies related to endometriosis, we covariated for surgery and adenomyosis, 2 conditions with potentially more pain and less responses to the treatment. We found that the results were equivalent, suggesting a similar effect in all subpopulations, including those with a more complex pathophysiology. Nevertheless, an effect in the range of 30% for Endocare can ultimately be considered moderately significant according to the IMMPACT guidelines [56]. This can be explained by the design of our study itself. Indeed, our relatively small sample size, or perhaps the unique use of the treatment, could have lessened the effect. Nevertheless, it is important to highlight the fact that we reached significance even with this design. Future studies should aim to confirm these results with a larger population size and repeated use of the treatment.

### Conclusions

In conclusion, this pilot study on the Endocare treatment shows encouraging results for developing a digital therapy to relieve patients of pelvic and perineal pain associated with endometriosis. Moreover, Endocare treatment can be a great alternative to hormonal treatment or surgery for women who wish to get pregnant. These results will be further investigated in a second study evaluating the analgesic effects of the repeated use of Endocare at home with a larger population of women living with chronic pelvic pain associated with endometriosis. In the future, Endocare could benefit patients diagnosed with endometriosis during their everyday life to reduce the acute and chronic pain encountered in this pathology, thus improving their quality of life.

## Appendix

Multimedia Appendix 1 CONSORT eHEALTH checklist (V 1.6.1).

## Abbreviations

AE: adverse event
CPP: chronic pelvic pain
DTx: digital therapeutics
FDR: false discovery rate
IFEMEndo: Franco-European Multidisciplinary Endometriosis Institute
IMMPACT: Initiative on Methods, Measurement, and Pain Assessment in Clinical Trials
MRI: magnetic resonance imagery
NRS: numerical rating scale
RCT: randomized controlled trial
VR: virtual reality

## References

[1] Vercellini P, Viganò P, Somigliana E, Fedele L (2014). Endometriosis: pathogenesis and treatment. Nat Rev Endocrinol.

[2] Saunders PTK, Horne AW (2021). Endometriosis: Etiology, pathobiology, and therapeutic prospects. Cell.

[3] Taylor HS, Kotlyar AM, Flores VA (2021). Endometriosis is a chronic systemic disease: clinical challenges and novel innovations. Lancet.

[4] Giudice LC (2010). Clinical practice. Endometriosis. N Engl J Med.

[5] Tirlapur S A, Kuhrt K, Chaliha C, Ball E, Meads C, Khan K S (2013). The 'evil twin syndrome' in chronic pelvic pain: a systematic review of prevalence studies of bladder pain syndrome and endometriosis. Int J Surg.

[6] Guo S, Wang Y (2006). The prevalence of endometriosis in women with chronic pelvic pain. Gynecol Obstet Invest.

[7] Dydyk A, Gupta N (2022). Chronic Pelvic Pain.

[8] Garcia LM, Birckhead BJ, Krishnamurthy P, Sackman J, Mackey IG, Louis RG, Salmasi V, Maddox T, Darnall BD (2021). An 8-week self-administered at-home behavioral skills-based virtual reality program for chronic low back pain: double-blind, randomized, placebo-controlled trial conducted during COVID-19. J Med Internet Res.

[9] Carbone MG, Campo G, Papaleo E, Marazziti D, Maremmani I (2021). The Importance of a Multi-Disciplinary Approach to the Endometriotic Patients: The Relationship between Endometriosis and Psychic Vulnerability. J Clin Med.

[10] Mechsner S (2021). Management of endometriosis pain : Stage-based treatment strategies and clinical experience. Schmerz.

[11] Vercellini P, Buggio L, Frattaruolo MP, Borghi A, Dridi D, Somigliana E (2018). Medical treatment of endometriosis-related pain. Best Pract Res Clin Obstet Gynaecol.

[12] Wattier J (2018). Conventional analgesics and non-pharmacological multidisciplinary therapeutic treatment in endometriosis: CNGOF-HAS Endometriosis Guidelines. Gynecol Obstet Fertil Senol.

[13] Kold M, Hansen T, Vedsted-Hansen H, Forman A (2012). Mindfulness-based psychological intervention for coping with pain in endometriosis. Nord Psychol.

[14] Jia S, Leng J, Shi J, Sun P, Lang J (2012). Health-related quality of life in women with endometriosis: a systematic review. J Ovarian Res.

[15] Kennedy S, Bergqvist A, Chapron C, D'Hooghe T, Dunselman G, Greb R, Hummelshoj L, Prentice A, Saridogan E (2005). ESHRE guideline for the diagnosis and treatment of endometriosis. Hum Reprod.

[16] Ball E, Khan KS (2020). Recent advances in understanding and managing chronic pelvic pain in women with special consideration to endometriosis. F1000Res.

[17] Meissner K, Schweizer-Arau A, Limmer A, Preibisch C, Popovici R, Lange I, de Oriol B, Beissner F (2016). Psychotherapy with somatosensory stimulation for endometriosis-associated pain: a randomized controlled trial. Obstet Gynecol.

[18] Dang A, Arora D, Rane P (2020). Role of digital therapeutics and the changing future of healthcare. J Family Med Prim Care.

[19] Bordeleau M, Stamenkovic A, Tardif P, Thomas J (2022). The Use of Virtual Reality in Back Pain Rehabilitation: A Systematic Review and Meta-Analysis. J Pain.

[20] Chow H, Hon J, Chua W, Chuan A (2021). Effect of virtual reality therapy in reducing pain and anxiety for cancer-related medical procedures: a systematic narrative review. J Pain Symptom Manage.

[21] Chuan A, Zhou JJ, Hou RM, Stevens CJ, Bogdanovych A (2021). Virtual reality for acute and chronic pain management in adult patients: a narrative review. Anaesthesia.

[22] Ding L, Hua H, Zhu H, Zhu S, Lu J, Zhao K, Xu Q (2020). Effects of virtual reality on relieving postoperative pain in surgical patients: A systematic review and meta-analysis. Int J Surg.

[23] López-Valverde N, Muriel-Fernández J, López-Valverde A, Valero-Juan LF, Ramírez JM, Flores-Fraile J, Herrero-Payo J, Blanco-Antona LA, Macedo-de-Sousa B, Bravo M (2020). Use of virtual reality for the management of anxiety and pain in dental treatments: systematic review and meta-analysis. J Clin Med.

[24] Mallari B, Spaeth EK, Goh H, Boyd BS (2019). Virtual reality as an analgesic for acute and chronic pain in adults: a systematic review and meta-analysis. JPR.

[25] Boyle Y, Bentley D, Watson A, Jones A (2006). Acoustic noise in functional magnetic resonance imaging reduces pain unpleasantness ratings. Neuroimage.

[26] Ecsy K, Jones A, Brown C (2017). Alpha-range visual and auditory stimulation reduces the perception of pain. Eur J Pain.

[27] Farzaneh M, Abbasijahromi A, Saadatmand V, Parandavar N, Dowlatkhah HR, Bahmanjahromi A (2019). Comparative effect of nature-based sounds intervention and headphones intervention on pain severity after cesarean section: a prospective double-blind randomized trial. Anesth Pain Med.

[28] Gerlinger C, Schumacher U, Faustmann T, Colligs A, Schmitz H, Seitz C (2010). Defining a minimal clinically important difference for endometriosis-associated pelvic pain measured on a visual analog scale: analyses of two placebo-controlled, randomized trials. Health Qual Life Outcomes.

[29] Facchin F, Barbara G, Saita E, Mosconi P, Roberto A, Fedele L, Vercellini P (2015). Impact of endometriosis on quality of life and mental health: pelvic pain makes the difference. J Psychosom Obstet Gynaecol.

[30] Arendsen LJ, Henshaw J, Brown CA, Sivan M, Taylor JR, Trujillo-Barreto NJ, Casson AJ, Jones AKP (2020). Entraining alpha activity using visual stimulation in patients with chronic musculoskeletal pain: a feasibility study. Front Neurosci.

[31] Terho A, Puhto T, Laru J, Uimari O, Ohtonen P, Rautio T, Koivurova S (2022). Laparoscopically guided transversus abdominis plane block versus local wound analgesia in laparoscopic surgery for peritoneal endometriosis: study protocol for a prospective randomized controlled double-blinded LTAP-trial. Trials.

[32] Dworkin RH, Turk DC, Farrar JT, Haythornthwaite JA, Jensen MP, Katz NP, Kerns RD, Stucki G, Allen RR, Bellamy N, Carr DB, Chandler J, Cowan P, Dionne R, Galer BS, Hertz S, Jadad AR, Kramer LD, Manning DC, Martin S, McCormick CG, McDermott MP, McGrath P, Quessy S, Rappaport BA, Robbins W, Robinson JP, Rothman M, Royal MA, Simon L, Stauffer JW, Stein W, Tollett J, Wernicke J, Witter J, IMMPACT (2005). Core outcome measures for chronic pain clinical trials: IMMPACT recommendations. Pain.

[33] Bermo M, Patterson D, Sharar S, Hoffman H, Lewis D (2020). Virtual reality to relieve pain in burn patients undergoing imaging and treatment. Top Magn Reson Imaging.

[34] Frey DP, Bauer ME, Bell CL, Low LK, Hassett AL, Cassidy RB, Boyer KD, Sharar SR (2019). Virtual reality analgesia in labor. Anesth Analg.

[35] Guo C, Deng H, Yang J (2015). Effect of virtual reality distraction on pain among patients with hand injury undergoing dressing change. J Clin Nurs.

[36] Pourmand A, Davis S, Marchak A, Whiteside T, Sikka N (2018). Virtual reality as a clinical tool for pain management. Curr Pain Headache Rep.

[37] Mosso-Vázquez JL, Gao K, Wiederhold BK, Wiederhold MD (2014). Virtual reality for pain management in cardiac surgery. Cyberpsychol Behav Soc Netw.

[38] JahaniShoorab N, Ebrahimzadeh Zagami S, Nahvi Ali, Mazluom SR, Golmakani N, Talebii M, Pabarja F (2015). The effect of virtual reality on pain in primiparity women during episiotomy repair: a randomize clinical trial. Iran J Med Sci.

[39] Jin W, Choo A, Gromala D, Shaw C, Squire P (2016). A virtual reality game for chronic pain management: a randomized, controlled clinical study. Stud Health Technol Inform.

[40] Jones T, Moore T, Choo J (2016). The impact of virtual reality on chronic pain. PLoS One.

[41] Wiederhold BK, Gao K, Sulea C, Wiederhold MD (2014). Virtual reality as a distraction technique in chronic pain patients. Cyberpsychol Behav Soc Netw.

[42] Osumi M, Ichinose A, Sumitani M, Wake N, Sano Y, Yozu A, Kumagaya S, Kuniyoshi Y, Morioka S (2017). Restoring movement representation and alleviating phantom limb pain through short-term neurorehabilitation with a virtual reality system. Eur J Pain.

[43] Ichinose A, Sano Y, Osumi M, Sumitani M, Kumagaya S, Kuniyoshi Y (2017). Somatosensory feedback to the cheek during virtual visual feedback therapy enhances pain alleviation for phantom arms. Neurorehabil Neural Repair.

[44] Moore R, Derry S, McQuay HJ, Wiffen P (2011). Single dose oral analgesics for acute postoperative pain in adults. Cochrane Database Syst Rev.

[45] Casciaro F, Laterza V, Conte S, Pieralice M, Federici A, Todarello O, Orsucci F, Conte E (2013). Alpha-rhythm stimulation using brain entrainment enhances heart rate variability in subjects with reduced HRV. WJNS.

[46] Le Scouarnec RP, Poirier RM, Owens JE, Gauthier J, Taylor AG, Foresman PA (2001). Use of binaural beat tapes for treatment of anxiety: a pilot study of tape preference and outcomes. Altern Ther Health Med.

[47] Pickens TA, Khan SP, Berlau DJ (2019). White noise as a possible therapeutic option for children with ADHD. Complement Ther Med.

[48] Farokhnezhad Afshar P, Bahramnezhad F, Asgari P, Shiri M (2016). Effect of white noise on sleep in patients admitted to a coronary care. J Caring Sci.

[49] Stanchina ML, Abu-Hijleh M, Chaudhry BK, Carlisle CC, Millman RP (2005). The influence of white noise on sleep in subjects exposed to ICU noise. Sleep Med.

[50] Papalambros NA, Santostasi G, Malkani RG, Braun R, Weintraub S, Paller KA, Zee PC (2017). Acoustic enhancement of sleep slow oscillations and concomitant memory improvement in older adults. Front Hum Neurosci.

[51] Zhou J, Liu D, Li X, Ma J, Zhang J, Fang J (2012). Pink noise: effect on complexity synchronization of brain activity and sleep consolidation. J Theor Biol.

[52] Saadatmand V, Rejeh N, Heravi-Karimooi M, Tadrisi SD, Zayeri F, Vaismoradi M, Jasper M (2013). Effect of nature-based sounds' intervention on agitation, anxiety, and stress in patients under mechanical ventilator support: a randomised controlled trial. Int J Nurs Stud.

[53] Tesarz J, Leisner S, Gerhardt A, Janke S, Seidler GH, Eich W, Hartmann M (2014). Effects of eye movement desensitization and reprocessing (EMDR) treatment in chronic pain patients: a systematic review. Pain Med.

[54] Bani Mohammad E, Ahmad M (2018). Virtual reality as a distraction technique for pain and anxiety among patients with breast cancer: A randomized control trial. Palliative & Supportive Care.

[55] Persky S, Lewis M (2019). Advancing science and practice using immersive virtual reality: what behavioral medicine has to offer. Transl Behav Med.

[56] Dworkin RH, Turk DC, Wyrwich KW, Beaton D, Cleeland CS, Farrar JT, Haythornthwaite JA, Jensen MP, Kerns RD, Ader DN, Brandenburg N, Burke LB, Cella D, Chandler J, Cowan P, Dimitrova R, Dionne R, Hertz S, Jadad AR, Katz NP, Kehlet H, Kramer LD, Manning DC, McCormick C, McDermott MP, McQuay HJ, Patel S, Porter L, Quessy S, Rappaport BA, Rauschkolb C, Revicki DA, Rothman M, Schmader KE, Stacey BR, Stauffer JW, von Stein T, White RE, Witter J, Zavisic S (2008). Interpreting the clinical importance of treatment outcomes in chronic pain clinical trials: IMMPACT recommendations. J Pain.

